# Return to Work during the COVID-19 Outbreak: A Study on the Role of Job Demands, Job Resources, and Personal Resources upon the Administrative Staff of Italian Public Universities

**DOI:** 10.3390/ijerph19041995

**Published:** 2022-02-10

**Authors:** Gloria Guidetti, Daniela Converso, Domenico Sanseverino, Chiara Ghislieri

**Affiliations:** 1 Department of Psychological Sciences, Health and Territory, University of G. d’Annunzio Chieti and Pescara, Via dei Vestini 31, 66100 Chieti, Italy; gloria.guidetti@unich.it; 2 Department of Psychology, University of Turin, Via Verdi 10, 10124 Turin, Italy; domenico.sanseverino@unito.it (D.S.); chiara.ghislieri@unito.it (C.G.)

**Keywords:** return to work, quantitative job demands, supervisor support, fatigue management, personal contribution in managing COVID-19, emotional exhaustion

## Abstract

Background: Compared to healthcare workers and teleworkers, occupational wellbeing of employees who continued or suddenly returned to work during the COVID-19 pandemic have received less attention thus far. Using the Job Demand–Resource model as a framework, the present study aimed at evaluating the role of job demands and job and personal resources in affecting emotional exhaustion among university administrative staff. Methods: This cross-sectional study analyzed data collected through an online questionnaire completed by 364 administrative employees that continued working in presence (WP) and 1578 that continued working blended (WB), namely, partly remotely and partly in presence. Results: Among job demands, quantitative job demand overloads and perceived risk of being infected were positively associated with higher levels of emotional exhaustion. Among job resources, colleague support was significantly associated with lower emotional exhaustion for both WB and WP, whereas supervisor support and fatigue management were salient only for WB. Among personal resources, personal contribution in managing COVID-19-related risk at work emerged as a protective factor for emotional exhaustion. Conclusion: Insights for the development of targeted preventive measure for a more psychologically safe and productive return to work can be derived from these results.

## 1. Introduction

The advent of the COVID-19 pandemic has radically changed the ways by which businesses have had to think about work processes and organization by putting health and safety management at the fore. From the earliest stages of the health emergency in March 2020, understanding the impact that the pandemic, as well as its occupational correlates, could have on workers’ well-being and mental health has been the subject of many studies in the field of work and organizational psychology (e.g., [1,2]). On the one side, to counter the spread of contagion, many public and private organizations “re-considered” new ways of working, such as tele-working and smart working practices, and a huge number of studies have been developed to understand risks and potentialities for remote workers’ productivity and wellbeing (e.g., [3,4,5]). On the other side, most of the focus has been placed on those occupational groups for which the degree of exposure to the risk of contagion and COVID-19-related challenges and demands turned the job conditions highly stressful, as is the case of the healthcare sector (e.g., [6,7,8,9]). However, as it has been pointed out by Rudolph and colleagues [10], focusing on health, safety, and wellbeing issues should be differentiated according to occupational groups. Despite this, less attention has been paid to those sectors that have been classified, on the basis of the levels of exposure, proximity, and aggregation, at medium-high level of contagion, such as public administration [11].

In Italy, since the implementation of lockdown measures on 11 March 2020, the public administration has introduced, to counteract the spread of contagion, forms of working from home, such as teleworking and smart working practices, that were mandatory for at least the 50% of employees. Compared to pre-pandemic period, a considerable expansion of remote work has been implemented. It has been estimated that about 58% of the employees in the Italian public administration sector worked, and are still working now, remotely [12]. However, some critical issues, primarily linked to difficulties in expanding digitalization tools and the management of the often and rigid bureaucratic procedures that distinguish this sector from the private one, hindered the adoption of remote working practices for the entirety of the staff. Therefore, since the earliest stages of the pandemic, and then more substantially starting from the second phase in May 2020, a considerable amount of public administration employees continued working in presence or started working in a mixed way, i.e., partly in presence and partly remotely.

On the one hand, this situation required organizations to deploy resources to ensure the physical and psychological safety of people facing a higher work-related risk of being infected and states of mental fatigue. Relatedly, in addition to the application of “technical” guidelines pertaining to engineering, administration, and use of PPE preventive measures [13], the relevance of considering psychosocial safety and “non-technical skills” has been recently outlined [14], by which both the effective application of technical measures and the perceived psychological safety and wellbeing depend [15,16,17]. On the other hand, such changes involved new ways of working that may significantly impact on future policies and culture shifts. As was recently pointed out [18,19], although mixed, or hybrid, working abruptly emerged due to the COVID-19 pandemic, it is likely that these forms of work will characterize the reorganizational plans not only in the short-, but also in the medium- and long-term, even in the public sector. This implies that organizations must consider how to deal with challenges and resource provision distinctly for workers who continue to work in presence and for those who are starting to work partly in presence and partly remotely.

In the light of these premises, this study aimed to address the need to increase research on COVID-19-related wellbeing and psychosocial safety among a currently overlooked working population, namely, that of administration staff of Italian public universities that suddenly returned working in presence, or has started in a mixed form, during the pandemic. To the best of our knowledge there is a lack of studies that currently have dealt with such issues. This may represent a first step in the process of understanding the encountered challenges and providing public organizations first indications for targeted interventions.

To achieve this goal, the hypotheses of the present study were developed according to the Job Demands–Resources model (JD-R) [20] that suggests that work conditions, which can be categorized into job demands and job resources, may affect employees’ health and wellbeing. Job demands refer to the physical, psychological, or socio-organizational aspects of the work, whose energy-depleting process induces people to experience energy loss and fatigue, leading to stress, burnout, and health impairment. Conversely, job resources refer to the physical, psychological, social, or organizational aspects of the job that reduce job demands and stress reactions while stimulating work motivation, personal growth, and development. In addition, personal resources have been introduced in the JD-R model defining them as “aspects of the self that are generally linked to resilience and refer to individuals’ sense of their ability to control and impact upon their environment successfully” ([21] p. 123), thus stimulating optimal functioning and lessening stress. Moreover, according to the JD-R model [20], any occupation and work environment have their own specific job demands and job resources. Hence, the present study considered some job demands and job resources and one personal resource that can be relevant in reference to wellbeing and psychosocial safety at work among public universities’ administration staff by investigating their relationship with employees’ stress reaction. Specifically, we consider the relationship with emotional exhaustion, as well as considering the distinction between those who continued to work in presence (WP) and those who started working in a blended way (WB).

### Literature Review and Hypothesis Development

One of the most influential factors that have been investigated in relation to wellbeing and mental health outcomes during the COVID-19 pandemic is the perceived risk of being infected. Generally, it can be viewed as a cognitive process involved in various daily activities, fostering people’s preventive and protective behavior when they must take decisions involving potential risks, such as with COVID-19 infection [22,23]. However, it can also have negative consequences on mental wellbeing by enhancing negative emotional responses and concerns. Referring to the occupational context, most of the research has primarily investigated front-line and healthcare workers by evidencing its association with psychological distress outcomes [24,25,26,27]. Studies have also highlighted that among workers of different productive and service jobs, perceiving return to work as a health risk was associated to higher levels of psychological distress [28,29]. Furthermore, it has been recently suggested that the perceived risk of being infected at work, while potentially increasing protective behaviors, may induce a psychological resources’ depleting process, thus increasing levels of exhaustion [17]. Following these lines of research and according to the J D-R model [20], the perceived risk of being infected in the workplace can then be conceptualized as a salient new psychosocial risk even among those workers of the public administration sector who continued working in presence or returned to work partly in presence and partly remotely since the first phases of the pandemic. The following hypothesis was formulated:

**Hypothesis** **1** **(H1).** *The perceived risk of being infected is positively associated with emotional exhaustion.*

Furthermore, it can be hypothesized that for WPs, the perception of being infected is greater compared to WBs due to the longer time spent in presence and the increase in social interactions within the workplace. In this vein, we can sustain that, despite being relevant for both sub-samples, the relationship between the perceived risk of being infected and emotional exhaustion will be stronger among WPs.

According to Kniffin and colleagues [30], there is variation across and within occupational sectors with respect to how COVID-19 has affected the demands associated with various jobs; furthermore, there is evidence suggesting that working conditions have deteriorated for most employees [31]. Therefore, along with the perceived risk of being infected, another job demand that may affect public administrative employees’ wellbeing can be represented by quantitative job demand overload. Quantitative, or psychological, demands are those relating to the “amount of work to be done” ([32] p. 308). A range of words that connotes high quantitative job demands includes work intensity, work pressure, or work overload; in turn, all of these expressions specifically imply sustained cognitive effort that, according to the JD-R model [20], are associated with higher levels of exhaustion. Although an increase in the workload has been recorded in both the healthcare sector and among those who shifted to remote working practices due to the pandemic emergency (e.g., [33,34,35]), this element can also be traced back to workers who, by returning to work, have had to readjust their job according to new procedures for managing the “new” or “next normal” [36] situation. Indeed, new routines and behaviors, such as wearing masks and social distancing, along with frequent messages about handwashing and cleaning, as well as new ways of working with physically distant colleagues, may have affected changes in work schedules; furthermore, these changes have been shown to increase stress, anxiety, and fatigue in general [31,37]. Thus, the following hypothesis was formulated:

**Hypothesis** **2** **(H2).** *Quantitative job demand overload is positively associated with emotional exhaustion.*

Despite no previous evidence being available, it is possible to hypothesize that, for WBs, the need to adapt to a mixed or hybrid work organization may involve a higher level of workload compared to WPs. In this vein, we can assume that despite being significant for both sub-samples, the relationship between quantitative job demand overload and emotional exhaustion will be stronger among WBs.

In relation to these circumstances, the centrality of being able to re-establish a balance through the use and deployment of specific resources was emphasized [30]. Through the employment of an integrated approach to safety, health, and wellbeing of workers who continued to work in presence during the pandemic, the centrality of work and organizational policies, programs, and practices that foster supportive conditions has been brought forth [16]. Among these, social support, which can be defined as the employee’s belief that help can be available from others in demanding conditions [38], has been investigated as one of the most powerful sources of wellbeing and mental health able to reduce uncertainties during unpredictable time of crisis. Therefore, it should have a further critical role, especially during the COVID-19 pandemic, when employees are under additional and atypical stress and are even more socially isolated [39,40]. In line with these arguments, recent studies have contended that among smart workers, both supervisor and co-worker support helped employees reduce emotional exhaustion by lessening perceived uncertainties and organizational crisis communication during the COVID-19 lockdown [41,42,43]. According to this evidence, it can be hypothesized that experiencing workplace social support, functional to the best management of difficulties caused by the pandemic emergency, may represent a significant workplace resource, even for those workers who had suddenly returned to work in presence or started to work partly in presence and partly remotely during the first phases of the COVID-19 pandemic. Thus, the following hypotheses were formulated:

**Hypothesis** **3a** **(H3a).** *Supervisor support is negatively associated with emotional exhaustion*.

**Hypothesis** **3b** **(H3b).** *Co-worker support is negatively associated with emotional exhaustion.*

Concerning these aspects, it is also possible to hypothesize that, due to higher social isolation and greater communication difficulties that WBs may face when working from home, the perceived supervisor and co-worker support could be lower among WBs compared to WPs. In this vein, it is possible to assume that despite being relevant for both subsamples, these resources may be more salient for WBs in the relationship with emotional exhaustion.

Along with social support experienced in vertical and horizontal workplace relationships, organizations should also provide resources able to recognize states of fatigue related to the context of uncertainty and radical change brought forth by the pandemic transition [31]. Fatigue is generally considered as a state of feeling tired, weary, or sleepy, one that results from prolonged mental and physical work, extended periods of anxiety, exposure to harsh environments, or loss of sleep [44], potentially being a precursor of chronic stress consequences such as burnout [45]. According to guidelines inherent to mitigation strategies to cope with workplace fatigue in times of COVID-19 [31], the role of fatigue management practices has recently been gaining recognition. Fatigue management can be referred to the identification of antecedents and consequences of fatigue (mental and physical) related to COVID-19 at work, as well as to the implementation of coping strategies [15]. Following the JD-R model [20], fatigue management practices can therefore be considered as a job resource able to lessen levels of emotional exhaustion. Thus, the following hypothesis was formulated:

**Hypothesis** **4** **(H4).** *Fatigue management is negatively associated with emotional exhaustion.*

Specifically, we can assume that organizational resources able to counteract states of fatigue may be effective for both WPs and WBs, and it is possible to hypothesize that there are no significant differences between the two subsamples in the perception of this organizational resource. However, it could be hypothesized that, since WBs may probably face more challenging changes and social isolation, organizational resources such as fatigue management practices may be more salient in the relationship with emotional exhaustion for WBs.

Finally, in order for worker safety, health, and wellbeing to be supported during the COVID-19 pandemic, an integrated and participatory approach for the enforcement of the identified procedures has been emphasized [16]. The broader literature on workplace safety has outlined the role of employees’ safety behaviors and safety participation, defined by acts such as helping co-workers, promoting the safety program, and suggesting changes [46] that help preventing accident and injuries as well as preserving health (e.g., [47,48]). These lines of research highlight the role of workers’ personal contribution in managing the COVID-19 risk at work, i.e., how much employees feel able to adopt functional behaviors to facilitate and improve all measures to combat contagion [15]. Therefore, according to the JD-R model [20], the following hypothesis was formulated:

**Hypothesis** **5** **(H5).** *personal contribution in managing COVID-19-related risk at work can be considered as a relevant personal resource that may lessen levels of emotional exhaustion among employees who continued, or suddenly returned, to work during the pandemic*.

It is possible to hypothesize that such proactive behaviors to safety may be higher among the WPs who must manage daily work in presence and for which the responsibility for their behavior may be more salient in safeguarding their health, not only physical but also psychological, compared to WBs.

## 2. Materials and Methods

### 2.1. Procedure and Participants

The study is part of a wider research intervention project of the National Conference of Equality in Italian Universities; the conference brings together, in a network, the representatives of the university committees working on equal opportunities and wellbeing issues, in order to build systematic inter-university cooperation relations on the areas of gender equality, work–life balance, wellbeing, and inclusion.

The data used for the purposes of the present study were collected among the administrative sector of these organizations by means of an online questionnaire. The respondents participated to the survey during working hours and no incentives were offered for participation in the survey. The survey, approved by the Bioethics Committee of the University of Turin on 22 October 2020 (prot. no. 458997), was proposed to the member universities who were provided with documents and informative materials to support the presentation of the study. Prior to the start of the survey, each university informed their various stakeholders (governance, management, and trade unions) by their own accord and procedures. The voluntary participation and anonymity of the participants were ensured, and all the ethical guidelines were followed according to the Declaration of Helsinki (and following revisions) and Italian regulations on data protection and privacy (Law 196/2003). At the end of the survey, the results were shared with the member universities through a presentation and discussion workshop, which also aimed to identify needs and possible interventions to be implemented at a general level. Subsequently, the universities received a detailed report that covered every investigated area in depth, consistent with an intervention-oriented research approach.

### 2.2. Measures

Exhaustion was assessed with the 8-item scale of the Oldenburg Burnout Inventory [49] (e.g., “After my work, I regularly feel worn out and weary”). The scale ranged from 1 (strongly disagree) to 5 (strongly agree). Cronbach’s alpha = 0.84.

Quantitative job demands were measured using 4 items adapted from Bakker and colleagues ([50,51] (i.e., time pressure, working fast, concentration load, mental demands). Respondents chose their answer on a 5-point Likert scale ranging from 1 (never) to 5 (always). Cronbach’s alpha = 0.80.

Perceived probability of being infected during daily work was assessed using 1 item (“What is the likelihood that you can be infected by COVID-19 in your work organization?”). Responses were measured on a 10-point Likert scale ranging from 1 (not at all) to 10 (completely).

Support from colleagues (“How much do you feel you can count on the support of your colleagues in this phase of the COVID-19 emergency?”) and from the supervisor (“How much do you feel you can count on the support of your direct supervisor in this phase of the COVID-19 emergency?”) were measured on a 10-point Likert scale ranging from 1 (not at all) to 10 (completely). These two questions were ad hoc measures developed for the present study.

Organizations’ COVID-19 fatigue management (e.g., “In order to contain contagion risks by COVID-19, it is important that each employee adopts specific behaviors, i.e., using PPE, keeping interpersonal distance, practicing remote working. In your opinion, your employer organization is able to recognize the possible effects of such behaviors on physical fatigue”) was assessed using the specific 4-item scales of the SAPH@work questionnaire [15]. Cronbach’s alpha = 0.97.

Finally, personal contribution to healthy and safe practices in relation to COVID-19 was assessed using the specific 5-item scale of the SAPH@work questionnaire [15]. Cronbach’s alpha = 0.86. SAPH@work scales used a 5-point Likert scale ranging from 1 (not at all) to 5 (completely). Reliability of the scales were satisfactory and above the threshold of 0.70.

### 2.3. Data Analysis

Data analyses were performed using SPSS (SPSS Statistics for Windows, Version 27.0., IBM Corp, Armonk, NY, USA), Version 27. Before proceeding with the analyses, as data were collected among 22 universities, the between-unit variability of individual perception of emotional exhaustion was calculated running mixed models with SPSS. Level 2 variance estimate (σ^2^μ_0j_) was equal to 0.0027, and not significant (*p* = 0.25). Therefore, we decided to ignore the nested structure of the data due to the low between-unit variability.

For each scale of the questionnaire, we calculated synthetic indexes; we then carried out descriptive analyses (mean (M) and standard deviation (SD)). Analysis of variance (ANOVA) was performed to detect differences on the study variables between groups according to work arrangement (continuing to work in presence or just partly). Differences according to gender and frequency of contact with service users were also evaluated for both WPs and WBs to understand if they could be relevant control variables to insert within the regression models. Pearson’s correlations were performed to detect relationship between variables, and hierarchical multiple regression models were established to evaluate the association between job demands, job and personal resources, and exhaustion.

## 3. Results

A total of 533 university administrative employees that continued working in presence (WP) and 2407 that continued working blended (WB), namely, partly remotely and partly in presence, agreed to participate in the survey. The present study was run on a sample of 364 WP and 1578 WB that correctly filled out the questionnaire. The WP sample was composed of a majority of females (65.4%) with a mean length of service of 20.5 years, and most of them had a permanent (93%) and full-time working hours (92.2%) contract. The majority (68.4%) had a master’s degree, whereas 26.1% had a high school diploma and 4.1% had a middle school diploma. The majority of people were employed in the research services area (30.9%), followed by educational (28.3%) and administrative procedure (14.1%) services. The remaining part of the sample was employed in human resources (9.3%), ICT (8.2%), logistics and maintenance (5.2%), internationalization (1.9%), finance (1.9%), and legal affairs (0.4%).

Among WPs, 62.6% declared that working in presence was their actual optimal working condition, 36% declared that their optimal working condition would have been working partly remotely and partly in presence, and only 1.4% declared that the optimal working condition would be remote work only. In the WP sample, 37.9% of the employees declared they had sporadic contact with service users, and 31.3% reported having frequent contact with users, whereas another 30.5% declared having no contact with users but only with colleagues.

The WB sample was composed of a majority of females (70.4%) as well, with a mean length of service of 17.8 years, and most of them had a permanent (90.4%) and full-time working hours (87.7%) contract. The majority (70.2%) had a master’s degree, whereas 26.8% had a high school diploma and 1.5% had a middle school diploma. The majority of people were employed in the educational services area (31.9%), followed by research (28.3%) and administrative procedure (19.2%) areas. The remaining part of the sample was employed in ICT (8.9%), human resources (6.5%), logistics and maintenance (4.8%), finance (5.1%), internationalization (2.9%), and legal affairs (0.6%).

Among WBs, 77.9% declared that working blended was the actual optimal working condition, whereas 10.8% declared that the optimal working condition would have been working completely in presence, and another smaller part (11.3%) stated that they wanted to work completely remotely. In the WB sample, 40.9% of the employees declared they had sporadic contact with users, and 26% reported having frequent contact with users, whereas the 33% declared having no contact with users but only with colleagues.

Table 1 and Table 2 display means, standard deviations, and Pearson’s correlations among the study variables for the WP and WB samples, respectively. All the variables correlated significantly and in the expected direction in relation with exhaustion. In both samples, age was significantly and negatively associated to colleague support: older workers experienced less colleague support in relation to COVID-19 management. In the WB sample, age was also significantly and negatively associated to fatigue management and personal contribution. No significant correlation emerged between age and exhaustion.

Furthermore, ANOVA analysis evidenced significant differences between the WP and WB levels of perceived probability of being infected by COVID-19 in daily work since WP reported lower levels compared to WB employees (F = 9.52, *p* = 0.002; M_WP_ = 5.04; M_WB_ = 5.47) (Table 3). This difference may be read in the light of the higher percentage of employees in the WB sample that stated that they wanted to work completely remotely (WB1) (11.3%). These employees indeed reported significant higher levels of perceived probability of being infected by COVID-19 in daily work compared to those who declared that working blended was their actual optimal working condition (WB2), and those who declared that the optimal working condition would have been working completely in presence (WB3) (F = 37.17, *p* < 0.001; M_WB1_ = 6.79; M_WB2_ = 5.36; M_WB3_ = 4.79).

Among the two samples, differences have been detected regarding gender (Table 4). In the WP sample, females reported significantly higher levels of quantitative job demand overload (F = 12.25, *p* < 0.001; M_WOMEN_ = 3.85; M_MEN_ = 3.53), whereas in the WB samples, females reported higher levels on quantitative job demand overload (F = 11.86, *p* < 0.001; M_WOMEN_ = 3.74; M_MEN_ = 3.60) and exhaustion (F = 10.00, *p* = 0.002; M_WOMEN_ = 2.84; M_MEN_ = 2.59), as well as lower levels of personal contribution (F = 12.25, *p* < 0.001; M_WOMEN_ = 3.64; M_MEN_ = 3.77).

Differences also emerged regarding contact with service users (Table 5). Specifically, in the WP sample, perceived probability of being infected by COVID-19 in daily work was significantly higher among those employees with frequent contact with service users (FCU, M = 6.04) compared with employees who had no (NCU, M = 4.39) or sporadic (SCU, M = 4.73) contacts with service users (F = 15.30, *p* < 0.001). In the WB sample, perceived probability of being infected by COVID-19 in daily work was significantly higher among FCU (M = 6.28) compared with NCU (M = 5.03) or SCU (M = 5.30) (F = 35.21, *p* < 0.001). Moreover, among the WB sample, it has also been evidenced that exhaustion was significantly higher among FCU (M = 2.87) compared with NCU (M = 2.71) or SCU (M = 2.74) (F = 15.30, *p* < 0.001). In the same vein, quantitative job demand overload was also significantly higher among FCU (M = 3.79) compared with NCU (M = 3.69) or SCU (M = 3.65) (F = 4.64, *p* = 0.026).

Table 6 and Table 7 display hierarchical regressions results for the WP and WB samples, respectively.

In the WP sample, in the first step, both quantitative job demand overload and perceived probability of being infected were positively associated with exhaustion. In the second step, colleague support, but not supervisor support, was significantly and negatively associated to exhaustion, along with quantitative job demands and perceived probability of being infected. In the third step, fatigue management was significantly and negatively associated with exhaustion, along with quantitative job demand overload, perceived probability of being infected, and colleague support. In the fourth step, personal contribution to healthy and safe practices in relation to COVID-19 management was entered by evidencing its negative and significant association with exhaustion. After inserting this variable, fatigue management stopped being significant, whereas both quantitative demands, perceived probability of being infected and colleague support, were still significantly associated with exhaustion. No control variables were inserted, since no significant differences concerning exhaustion were evidenced regarding gender, age, and frequency of service user contact.

In the WB sample, in the first step, quantitative job demand overload and perceived probability of being infected were positively associated with exhaustion. In the second step, both colleague support and supervisor support were significantly and negatively associated with exhaustion, along with quantitative job demand overload. At this step, perceived probability of being infected was no longer significant. In the third step, fatigue management resulted in being significantly and negatively associated with exhaustion, along with quantitative job demand overload and support from colleagues and the supervisor. In the fourth step, personal contribution to healthy and safe practices in relation to COVID-19 was entered, evidencing its negative and significant association with exhaustion. In the fifth step, control variables were entered, evidencing that being female was associated with higher levels of exhaustion, whereas the frequency of contact with service users evidenced no significant association with exhaustion.

The changing in R^2^ was significant for all the steps, both in the WP and WB regression models.

## 4. Discussion

The present study had the aim of investigating employees’ wellbeing in the university administration sector during the COVID-19 pandemic. Using the JD-R model as a framework [20], we addressed this issue by investigating the relationship between job demands and job and personal resources relevant for the specific work-related conditions of employees that continued to work in presence or that suddenly returned to work since the first phases of the pandemic. These employees, despite having to readjust to new ways of working and having to face a higher risk of being infected by COVID-19 in the workplace, have received scarce attention until now. This study thus contributes to the acknowledgement of factors that may influence a psychologically safe return to work and a better adaptation to the so-called “new normal”, which will affect workplace organization as long as the pandemic will persist.

Since our sample was composed of workers that continued working in presence (WP) or started working blended (WB), we ran analyses by considering differences between these two groups. ANOVA results evidenced that the only significant difference between the two sub-samples was detected regarding the perceived risk of being infected at work that, contrary to expectations, resulted in being higher among the WB sample compared to the WP sample. By a further inspection, we were able to hypothesize that this result was determined by the higher prevalence of workers in the WB sample in comparison to the WP, which stated that their optimal working condition would have been working completely remotely. This is consistent with past studies that evidenced that remote work can be a useful solution, especially for people concerned about COVID-19 [3,5].

However, no other significant differences have emerged regarding the quantitative job demand overload, nor the job and personal resources or the level of emotional exhaustion between WPs and WBs.

Another notable finding was detected regarding the frequency of contact with service users. Indeed, it emerged that those who had frequent contact with service users reported significantly higher levels of perceived risk of being infected at work in comparison with those with no contact or who had it sporadically. This is in accordance with previous studies in the mental health and healthcare sectors [52], strengthening the evidence of vulnerability for stress reactions, even for with having higher contact frequency with external users in non-healthcare services. Moreover, in the WB sample, having frequent contact with service users was also associated with higher levels of emotional exhaustion and quantitative job demand overload. This result highlights that working partly in presence and partly remotely can lead people to greater difficulties in the management of work tasks, especially when these involve frequent interaction with service users and being more vulnerable to the onset of stress-related symptoms. Gender differences also emerged. Consistently with previous studies [52,53,54], females reported higher levels of emotional exhaustion and psychological distress, as well as higher levels of perceived risk of being infected. In this regard, Sandín and colleagues [55] have suggested that there a is a gender difference in the psychological experience, somatization, and impact of the COVID-19 pandemic and the emotions it provokes, suggesting that women are more emotionally vulnerable to the effects of the COVID-19 context than men.

Finally, two distinct multiple regression models were run to detect the relationship between job demands and resources and emotional exhaustion for the WB and the WP samples.

First, the findings highlighted that quantitative job demand overload and the perceived risk of being infected at the workplace represent significant psychosocial risk factors. On the one side, the perceived risk of being infected at work was proven to be a salient job demand, capable of depleting psychological resources, as it was previously demonstrated [17], when considering the return to work during a pandemic period by negatively affecting psychological wellbeing. Specifically, the results of the present study evidenced that, despite the fact that the levels of perceived risk of being infected were higher among WBs, the regression results proved that this peculiar job demand, net of other variables, was significantly associated with higher levels of emotional exhaustion among WPs only. This is consistent with our hypothesis and previous evidence [3,5] on the protective role of working remotely for psychological safety by expanding the evidence that even a hybrid form of work can be a resource to preserve a higher psychological wellbeing in times of a pandemic.

On the other side, quantitative job demand overload emerged as a significant job demand in the relationship with emotional exhaustion for both WPs and WBs. Consistent with existing evidence from different occupations [31,33,34,35], we can confirm that employees of the public administrative sector that continued, or suddenly returned to work, since the first phases of the pandemic had to significantly bear an increase in job-related demands. This has probably been affected by new work procedures and schedules, as well as the need to manage work with remote colleagues and at the same time having to maintain and comply with the procedures for the prevention of contagion that involved work organization of both WPs and WBs.

Regarding job resources, differences between the two samples emerged. Indeed, if co-worker support resulted in being negatively associated with emotional exhaustion in both samples, for the WB sample, supervisor support and fatigue management were also significantly associated with lower exhaustion levels. This is coherent, on the one side, with the extant literature on the protective role of horizontal and vertical support experienced within the workplace, especially during the COVID-19 pandemic, when it is assumed that employees are under additional and atypical stress [41,42]. In this vein, we can support that working partly in presence and partly remotely may lead to employees feeling more socially isolated and requiring higher efforts on the level of coordination of activities; therefore, the role of the supervisor and the perception of the management’s skills resulted in being more salient. Such remarks may on the other hand explain the significant role of fatigue management practices only for the WB sample. For those who had to change their work practices in a hybrid way, perceiving support not only from colleagues and supervisors but also from the organization can be more relevant. Specifically, when such support is directed to the recognition of fatigue states and recovery needs, it can represent a salient job resource.

Finally, the findings of this study highlighted that, beyond the impact exerted from job resources, personal resources—specifically, the personal contribution of workers in managing the COVID-19 risk at work—are also negatively associated to exhaustion. This result has emerged for the WP sample, in which fatigue management stopped being significant after inserting the personal contribution in managing COVID-19 risk at work. In the WB sample, all the job resources remained significant after inserting the personal resource in the last step. We can identify the proactivity behavior toward safety and health at work as a form of engagement with the organization that in turn may limit the risk of exhaustion, especially in times of crisis and uncertainties that, on the contrary, may expose to a greater risk of stress and burnout. This is in accordance with recent qualitative studies conducted in the healthcare sector [34] that reported how proactivity, motivation, and personal initiatives were recognized as personal resources functional to sustain a better adaptation in the difficult times of the COVID-19 pandemic. However, future studies can evaluate more complex relationships between safety participation behaviors and stress reactions. For example, studies showed the moderating role of burnout on the relationship between safety participation and safety outcomes [56]. Moreover, studies may also expand such evidence by applying longitudinal research designs to analyze mutual relationships between emotional exhaustion and safety behaviors.

In this regard, one of the limitations of the present study is the absence of longitudinal data that would have allowed a measurement in different time points during the first year of the pandemic emergency and to control for cross-lagged association. Furthermore, the use of self-reported measurement may affect common method variance [57]. Future studies might utilize data from multiple sources (e.g., co-workers, supervisors) and control for multiple levels of information by employing multilevel analyses. Finally, since the data of the present study were collected from a non-randomized sample of higher education administrative employees, results cannot be generalized to other occupational populations that continued or abruptly returned to work during the pandemic. Research on these topics should therefore be implemented among different occupations by considering other specific job demands and resources that may have significantly affected health and wellbeing when returning to work.

Despite these limitations, the present study offers some reflections and practical implications concerning return to work and the management of the pandemic emergency within occupational sectors such as the public administration. Indeed, looking at both job demands, and job and personal resources may improve the focus on both job-related risk prevention and health-promoting strategies.

Organizations in the public administrative sector that are planning the “new normal” should consider the stressful potential of returning to work, for instance, by monitoring workloads through organizational actions of job design and involving employees through interventions that allow bottom-up work-related management, such as job-crafting [58]. Furthermore, employers should foster actions that can prevent not only the infection, but also the perception to infection exposure at the workplace. According to the present study, specific attention should be paid on how to manage COVID-19-related concerns among employees who return to work in presence. In this vein, secondary prevention strategies aimed at informing employees about COVID-19 health-related risk and organizational actions that have been adopted to effectively prevent contagion should be implemented.

On the other side, this study evidenced the need to pay attention and to sustain social participatory and support practices [59]. Considering the need of social distancing, organizations can support a better social climate between colleagues by providing remote psychosocial intervention at the group level aimed at analyzing group dynamics, improving relationships with colleagues, and improving group climate. Moreover, especially when designing hybrid forms of work, interventions should aim at sustaining leadership empowerment and strengthening leaders’ emotion regulation skills that can represent primary prevention strategies able to sustain employees’ need of supervisor support. At the same time, supportive organizations should recognize first symptoms of employee’s fatigue and how to manage them. Tertiary prevention strategies, such as psychological support through counselling actions, can be implemented to this aim.

Finally, organizational strategies should also support or strengthen personal resources to foster contribution of workers in managing COVID-19-related issues. Specifically, psycho-educational interventions that can sustain people motivation, proactivity, work meaning, and functional behaviors to cope with COVID-19 at the workplace may be effective for a psychologically safer return to work, both for WPs and for WBs.

## 5. Conclusions

This research approach, theoretically founded, systematic, and on a national scale, represents a working method that can enhance our general understanding of organizational phenomena while also granting a more in-depth reading in a “situated” perspective. This could allow universities and, more generally, public administrations, to reflect on the present situation (with an evidence-based approach) and to define ad hoc interventions addressed to the management and the protection of the health of employees. Future research directions can deepen the knowledge in this field by examining other factors that may be potentially impacting on the process of returning to work. These could include investigations concerning other types of job demands, such as technology use, role, and work–life conflict, as well as aspects that may influence employees needs of working in presence or in a hybrid way, such as quality of commuting or care burden. Moreover, design research methods such as a diary study might capture more accurate information on daily or weekly experiences that can be compared between WP and WB.

## Figures and Tables

**Table 1:** Means, standard deviations, Cronbach’s alphas, and correlations among the study variables in the sample of employees who continued working in presence.

	1	2	3	4	5	6	7	8
1. Age	1	0.099	−0.059	−0.05	−0.118 *	−0.062	0.047	−0.061
2. Exhaustion		1	0.282 **	0.195 **	−0.368 **	−0.253 **	−0.273 **	−0.232 **
3. Quantitative demands			1	0.112 *	−0.115 *	−0.082	−0.143 **	0.074
4. Work contagion				1	−0.193 **	−0.163 **	−0.055	−0.089
5. Co-worker support					1	0.569 **	0.405 **	0.267 **
6. Supervisor support						1	0.427 **	0.269 **
7. Fatigue management							1	0.248 **
8. Personal contribution								1

* *p* < 0.05; ** *p* < 0.01.

**Table 2:** Means, standard deviations, Cronbach’s alphas, and correlations among the study variables in the sub-sample of smart workers.

	1	2	3	4	5	6	7	8
1. Age	1	0.034	0.006	−0.002	−0.064 *	−0.023	−0.056 *	−0.052 *
2. Exhaustion		1	0.351 **	0.108 **	−0.279 **	−0.267 **	−0.225 **	−0.269 **
3. Quantitative demands			1	0.126 **	−0.064 *	−0.074 **	−0.149 **	−0.014
4. Work contagion				1	−0.160 **	−0.148 **	−0.032	0.019
5. Co-worker support					1	0.668 **	0.329 **	0.284 **
6. Supervisor support						1	0.449 **	0.246 **
7. Fatigue management							1	0.223 **
8. Personal contribution								1

* *p* < 0.05; ** *p* < 0.01.

**Table 3:** Analysis of variance to compare variables among WP vs. WB.

	WP	WB	F	*p*
	M (SD)	M (SD)		
Exhaustion	2.81 (0.76)	2.76 (0.73)	1.20	0.27
Quantitative demands	3.74 (0.83)	3.70 (0.77)	0.91	0.33
Work contagion	5.04 (2.4)	5.47 (2.4)	9.52	0.002
Co-worker support	7.01 (2.6)	7.28 (2.4)	3.47	0.06
Supervisor support	6.65 (2.95)	6.77 (2.84)	0.51	0.47
Fatigue management	2.72 (1.17)	2.67 (1.12)	0.49	0.48
Personal contribution	3.75 (0.78)	3.68 (0.74)	2.44	0.12

WP = Working in Presence; WB = Working Blended; M = mean; SD = standard deviation.

**Table 4:** Analysis of variance to compare variables according to gender among WPs and WBs.

WPs	Females (*n* = 238)	Males (*n* = 126)	F	*p*
	M (SD)	M (SD)		
Exhaustion	2.84 (0.79)	2.75 (0.72)	1.16	0.26
Quantitative demands	3.85 (0.84)	3.53 (0.78)	12.25	<0.001
Work contagion	5.06 (2.6)	4.98 (2.2)	0.82	0.77
Co-worker support	6.99 (2.6)	7.05 (2.5)	0.38	0.84
Supervisor support	6.53 (3.05)	6.88 (2.74)	1.19	0.27
Fatigue management	2.64 (1.18)	2.88 (1.13)	3.40	0.07
Personal contribution	3.72 (0.78)	3.80 (0.78)	0.92	0.33
**WBs**	**Females** **(*n* = 1111)**	**Males (*n* = 467)**	**F**	***p***
	**M (SD)**	**M (SD)**		
Exhaustion	2.84 (0.73)	2.59 (0.72)	36.7	<0.001
Quantitative demands	3.74 (0.77)	3.60 (0.75)	11.8	<0.001
Work contagion	5.54 (2.4)	5.29 (2.3)	3.73	0.053
Co-worker support	7.27 (2.6)	7.31 (2.4)	0.93	0.76
Supervisor support	6.72 (2.84)	6.89 (2.83)	1.24	0.26
Fatigue management	2.64 (1.11)	2.75 (1.13)	3.25	0.07
Personal contribution	3.64 (0.74)	3.77 (0.72)	10.00	0.002

M = mean; SD = standard deviation.

**Table 5:** Analysis of variance to compare variables according to contact with service users among WPs and WBs.

WPs	NCU (*n* = 112)	SCU (*n* = 138)	FCU (*n* = 114)	F	*p*
	M (SD)	M (SD)	M (SD)		
Exhaustion	2.81 (0.72)	2.81 (0.71)	2.83 (0.76)	0.20	0.98
Quantitative demands	3.81 (0.80)	3.66 (0.89)	3.77 (0.78)	1.16	0.31
Work contagion	4.39 (2.35)	4.73 (2.22)	6.05 (2.65)	15.30	<0.001
Co-worker support	7.33 (2.76)	7.12 (2.53)	6.61 (2.52)	2.37	0.10
Supervisor support	6.68 (3.0)	7.01 (2.84)	6.19 (2.90)	2.40	0.09
Fatigue management	2.68 (1.23)	2.73 (1.14)	2.74 (1.16)	1.00	0.90
Personal contribution	3.74 (0.76)	3.70 (0.83)	3.81 (74)	0.61	0.54
**WBs**	**NCU (*n* = 518)**	**SCU (*n* = 642)**	**FCU** **(*n* = 418)**	**F**	***p***
	**M (SD)**	**M (SD)**	**M (SD)**		
Exhaustion	2.71 (0.74)	2.74 (0.72)	2.87 (0.73)	5.89	0.003
Quantitative demands	3.65 (0.76)	3.69 (0.73)	3.79 (0.81)	3.64	0.02
Work contagion	5.03 (2.31)	5.30 (2.26)	6.28 (2.47)	35.21	<0.001
Co-worker support	7.45 (2.39)	7.32 (2.36)	6.99 (2.60)	4.29	0.014 *
Supervisor support	6.82 (2.76)	6.84 (2.74)	6.59 (2.95)	1.09	0.33
Fatigue management	2.67 (1.12)	2.69 (1.10)	2.65 (1.14)	0.20	0.81
Personal contribution	3.70 (0.76)	3.68 (0.83)	3.66 (74)	0.29	0.74

M = mean; SD = Standard Deviation; * = Leven’s test significant.

**Table 6:** Stepwise multiple regression parameters among employees who continued working in presence (WP sample).

		Beta	Standard Error
1			
	Quantitative job demands	0.263 ***	0.186
	Work contagion	0.166 ***	0.046
	R^2^ = 0.11 Δ R^2^ = 0.10		
2			
	Quantitative job demands	0.232 ***	0.221
	Work contagion	0.105 *	0.044
	Co-worker support	−0.292 ***	0.015
	Supervisor support	−0.051	0.017
	R^2^ = 0.21 Δ R^2^ = 0.10		
3			
	Quantitative job demands	0.22 ***	0.227
	Work contagion	0.111 *	0.044
	Co-worker support	−0.263 ***	0.015
	Supervisor support	−0.015	0.017
	Fatigue management	−0.123 *	0.015
	R^2^ = 0.22 Δ R^2^ = 0.01		
4			
	Quantitative job demands	0.24 ***	0.257
	Work contagion	0.104 *	0.044
	Co-worker support	−0.242 ***	0.015
	Supervisor support	0.004	0.017
	Fatigue management	−0.099	0.015
	Personal contribution	−0.153 **	0.035
	R^2^ = 0.24 Δ R^2^ = 0.02		

* *p* < 0.05; ** *p* < 0.01, *** *p* < 0.001.

**Table 7:** Stepwise multiple regression parameters among employees who worked partly remotely and partly in presence (WB sample).

		Beta	Standard Error
1			
	Quantitative job demands	0.344 ***	0.023
	Work contagion	0.061 **	0.007
	R^2^ = 0.13 Δ R^2^ = 0.13		
2			
	Quantitative job demands	0.329 ***	0.022
	Work contagion	0.018	0.007
	Co-worker support	−0.172 ***	0.009
	Supervisor support	−0.123 ***	0.008
	R^2^ = 0.20 Δ R^2^ = 0.07		
3			
	Quantitative job demands	0.319 ***	0.022
	Work contagion	0.023	0.007
	Co-worker support	−0.168 ***	0.009
	Supervisor support	−0.09 **	0.008
	Fatigue management	−0.082 ***	0.017
	R^2^ = 0.21 Δ R^2^ = 0.01		
4			
	Quantitative job demands	0.322 ***	0.022
	Work contagion	0.036	0.007
	Co-worker support	−0.124 ***	0.009
	Supervisor support	−0.079 **	0.008
	Fatigue management	−0.055 *	0.017
	Personal contribution	−0.198 ***	0.023
	R^2^ = 0.24 Δ R^2^ = 0.03		
5			
	Quantitative job demands	0.313 ***	0.022
	Work contagion	0.024	0.007
	Co-worker support	−0.126 ***	0.009
	Supervisor support	−0.079 **	0.008
	Fatigue management	−0.053 *	0.017
	Personal contribution	−0.189 ***	0.023
	Gender (male = 0)	0.10 ***	0.036
	Sporadic contact with service user	0.013	0.038
	Frequent contact with service user	0.04	0.044
	R^2^ = 0.25 Δ R^2^ = 0.02		

* *p* < 0.05; ** *p* < 0.01, *** *p* < 0.001.

## Data Availability

The data presented in this study are available on request from the corresponding author. The data are not publicly available due to the Italian law on privacy.

## References

[1] Nieuwenhuis R., Yerkes M.A. (2020). Workers’ well-being in the context of the first year of the COVID-19 pandemic. Community Work Fam..

[2] Ruiz-Frutos C., Ortega-Moreno M., Allande-Cussó R., Domínguez-Salas S., Dias A., Gómez- Salgado J. (2021). Health-related factors of psychological distress during the COVID-19 pandemic among non-health workers in Spain. Saf. Sci..

[3] Galanti T., Guidetti G., Zappalà S., Toscano F. (2021). Work from Home During the COVID-19 Outbreak: The Impact on Employees’ Remote Work Productivity, Engagement, and Stress. J. Occup. Environ. Med..

[4] Ghislieri C., Molino M., Dolce V., Sanseverino D., Presutti M. (2021). Work-family conflict during the COVID-19 pandemic: Teleworking of administrative and technical staff in healthcare: An Italian study. Med. Lav..

[5] Toscano F., Zappalà S. (2020). Social isolation and stress as predictors of productivity perception and remote work satisfaction during the COVID-19 pandemic: The role of concern about the virus in a moderated double mediation. Sustainability.

[6] Apaydin E.A., Rose D.E., Yano E.M., Shekelle P.G., McGowan M.G., Antonini T.L., Valdez C.A., Peacock M., Probst L., Stockdale S.E. (2021). Burnout Among Primary Care Healthcare Workers During the COVID-19 Pandemic. J. Occup. Environ. Med..

[7] Castelli L., Di Tella M., Benfante A., Taraschi A., Bonagura G., Pizzini A., Romeo A. (2021). The psychological impact of COVID-19 on general practitioners in Piedmont, Italy. J. Affect. Disord..

[8] Di Trani M., Mariani R., Ferri R., De Berardinis D., Frigo M.G. (2021). From resilience to burnout in healthcare workers during the COVID-19 emergency: The role of the ability to tolerate uncertainty. Front. Psychol..

[9] Giorgi G., Lecca L.I., Alessio F., Finstad G.L., Bondanini G., Lulli L.G., Arcangeli G., Mucci N. (2020). COVID- 19-Related Mental Health Effects in the Workplace: A Narrative Review. Int. J. Environ. Res. Public Health.

[10] Rudolph C.W., Allan B., Clark M., Hertel G., Hirschi A., Kunze F., Shockley K., Shoss M., Sonnentag S., Zacher H. (2021). Pandemics: Implications for Research and Practice in Industrial and Organizational Psychology. Ind. Organ. Psychol..

[11] Iavicoli S., Boccuni F., Buresti G., Gagliardi D., Persechino B., Valenti A., Rondonone B.A. (2021). Risk assessment at work and prevention strategies on COVID-19 in Italy. PLoS ONE.

[12] Osservatorio Smart Working. https://www.osservatori.net/it/ricerche/osservatori-attivi/smart-working.

[13] Dehghani F., Omidi F., Yousefinejad S., Taheri E. (2020). The hierarchy of preventive measures to protect workers against the COVID-19 pandemic: A review. Work.

[14] Mariani M.G., Vignoli M., Chiesa R., Violante F.S., Guglielmi D. (2019). Improving safety through non-technical skills in chemical plants: The validity of a questionnaire for the self-assessment of workers. Int. J. Environ. Res. Public Health.

[15] Converso D., Bruno A., Capone V., Colombo L., Falco A., Galanti T., Girardi D., Guidetti G., Viotti S., Loera B. (2021). Working during a Pandemic between the Risk of Being Infected and/or the Risks Related to Social Distancing: First Validation of the SAPH@W Questionnaire. Int. J. Environ. Res. Public Health.

[16] Dennerlein J.T., Burke L., Sabbath E.L., Williams J.A.R., Peters S.E., Wallace L., Karapanos M., Sorensen G. (2020). An Integrative Total Worker Health Framework for Keeping Workers Safe and Healthy During the COVID-19 Pandemic. Hum. Factors.

[17] Falco A., Girardi D., Dal Corso L., Yıldırım M., Converso D. (2021). The perceived risk of being infected at work: An application of the job demands–resources model to workplace safety during the COVID-19 outbreak. PLoS ONE.

[18] OECD The Future of (Remote?) Work in the Public Service: Five Ways to Find a Balance between Remote and In-Office Presence in the Public Service. https://www.oecd.org/gov/pem/finding-a-new-balance-between-remote-and-in-office-presence.pdf.

[19] Eures The Future of Work Is Hybrid: Blending Work from Different Locations. https://ec.europa.eu/eures/public/future-work-hybrid-blending-work-different-locations-2021-11-04_en.

[20] Bakker A.B., Demerouti E. (2017). Job demands–resources theory: Taking stock and looking forward. J. Occup. Health Psychol..

[21] Schaufeli W.B., Taris T.W., Bauer G.F., Hämmig O. (2014). A Critical Review of the Job Demands-Resources Model: Implications for Improving Work and Health. Bridging Occupational, Organizational and Public Health.

[22] Capone V., Caso D., Donizzetti A.R., Procentese F. (2020). University Student Mental Well-Being during COVID-19 Outbreak: What Are the Relationships between Information Seeking, Perceived Risk and Personal Resources Related to the Academic Context?. Sustainability.

[23] Wise T., Zbozinek T.D., Michelini G., Hagan C.C., Mobbs D. (2020). Changes in risk perception and self-reported protective behaviour during the first week of the COVID-19 pandemic in the United States. R. Soc. Open Sci..

[24] Krok D., Zarzycka B. (2020). Risk perception of COVID-19, meaning-based resources and psychological well-being amongst healthcare personnel: The mediating role of coping. J. Clin. Med..

[25] Gorini A., Fiabane E., Sommaruga M., Barbieri S., Sottotetti F., La Rovere M.T., Tremoli E., Gabanelli P. (2020). Mental health and risk perception among Italian healthcare workers during the second month of the COVID-19 pandemic. Arch. Psychiatr. Nurs..

[26] Lai J., Ma S., Wang Y., Cai Z., Hu J., Wei N., Wu J., Du H., Chen T., Li R. (2020). Factors associated with mental health outcomes among health care workers exposed to coronavirus disease 2019. JAMA Netw. Open.

[27] Sasangohar F., Jones S.L., Masud F.N., Vahidy F.S., Kash B.A. (2020). Provider Burnout and Fatigue During the COVID-19 Pandemic: Lessons Learned from a High-Volume Intensive Care Unit. Anesth. Analg..

[28] Tan W., Hao F., McIntyre R.S., Jiang L., Jiang X., Zhang L., Zhao X., Zou Y., Hu Y., Luo X. (2020). Is returning to work during the COVID-19 pandemic stressful? A study on immediate mental health status and psychoneuroimmunity prevention measures of Chinese workforce. Brain Behav. Immun..

[29] Sasaki N., Kuroda R., Tsuno K., Kawakami N. (2020). Workplace responses to COVID-19 associated with mental health and work performance of employees in Japan. J. Occup. Health.

[30] Kniffin K.M., Narayanan J., Anseel F., Antonakis J., Ashford S.P., Bakker A.B., Bamberger P., Bapuji H., Bhave D.P., Choi V.K. (2021). COVID-19 and the workplace. Implications, Issues and Insights for future research and actions. Am. Psychol..

[31] Wong I., O’Connor M. (2021). COVID-19 and Workplace Fatigue: Lessons Learned and Mitigation Strategies. https://blogs.cdc.gov/niosh-science-blog/2021/01/13/covid-19-fatigue.

[32] Kristensen T.S., Bjorner J.B., Christensen K.B., Borg V. (2004). The distinction between work pace and working hours in the measurement of quantitative demands at work. Work Stress.

[33] Barello S., Caruso R., Palamenghi L., Nania T., Dellafiore F., Bonetti L., Silenzi A., Marotta C., Graffigna G. (2021). Factors associated with emotional exhaustion in healthcare professionals involved in the COVID-19 pandemic: An application of the job demands-resources model. Int. Arch. Occup. Environ. Health.

[34] Giusino D., De Angelis M., Mazzetti G., Christensen M., Innstrand S.T., Faiulo I.R., Chiesa R. (2021). “We All Held Our Own”: Job Demands and Resources at Individual, Leader, Group, and Organizational Levels During COVID-19 Outbreak in Health Care. A Multi-Source Qualitative Study. Workplace Health Saf..

[35] Ingusci E., Signore F., Giancaspro M.L., Manuti A., Molino M., Russo V., Zito M., Cortese C.G. (2021). Workload, Techno Overload, and Behavioral Stress During COVID-19 Emergency: The Role of Job Crafting in Remote Workers. Front. Psychol..

[36] Sneader K., Singhal S. Beyond Coronavirus: The Path to the Next Normal. https://www.mckinsey.com/industries/healthcare-systems-and-services/our-insights/beyond-coronavirus-the-path-to-the-next-normal.

[37] Wang C., Pan R., Wan X., Tan Y., Xu L., McIntyre R.S., Choo F.N., Tran B., Ho R., Sharma V.K. (2020). A longitudinal study on the mental health of general population during the COVID-19 epidemic in China. Brain Behav. Immun..

[38] Mayo M., Sanchez J.I., Pastor J.C., Rodriguez A. (2012). Supervisor and coworker support: A source congruence approach to buffering role conflict and physical stressors. J. Hum. Resour. Manag..

[39] Sinclair R.R., Allen T., Barber L., Bergman M., Britt T., Butler A., Ford M., Hammer L., Kath L., Probst T. (2020). Occupational Health Science in the Time of COVID-19: Now more than ever. Occup. Health Sci..

[40] Spagnoli P., Manuti A., Buono C., Ghislieri C. (2021). The Good, the Bad and the Blend: The Strategic Role of the “Middle Leadership” in Work-Family/Life Dynamics during Remote Working. Behav. Sci..

[41] Charoensukmongkol P., Phungsoothorn T. (2020). The Interaction Effect of Crisis Communication and Social Support on The Emotional Exhaustion of University Employees during the COVID-19 Crisis. Int. J. Bus. Commun..

[42] Charoensukmongkol P., Phungsoonthorn T. (2020). The effectiveness of supervisor support in lessening perceived uncertainties and emotional exhaustion of university employees during the COVID-19 crisis: The constraining role of organizational intransigence. J. Gen. Psychol..

[43] Usman M., Cheng J., Ghani U., Gul H., Shah W.U. (2021). Social support and perceived uncertainties during COVID-19: Consequences for employees’ wellbeing. Curr. Psychol..

[44] Sadeghniiat-Haghighi K., Yazdi Z. (2015). Fatigue management in the workplace. Ind. Psychiatry J..

[45] Zarei E., Khakzad N., Reniers G., Akbari R. (2016). On the relationship between safety climate and occupational burnout in healthcare organizations. Saf. Sci..

[46] Van Dyne L., LePine J.A. (1998). Helping and voice extra-role behaviors: Evidence of construct and predictive validity. Acad. Manag. J..

[47] Curcuruto M., Parker S.K., Griffin M.A. (2019). Proactivity towards workplace safety improvement: An investigation of its motivational drivers and organizational outcomes. Eur. J. Work Organ. Psychol..

[48] Zohar D. (2008). Safety climate and beyond: A multi-level multi-climate framework. Saf. Sci..

[49] Demerouti E., Bakker A.B., Halbesleben J.R.B. (2008). The Oldenburg Burnout Inventory: A good alternative to measure burnout and engagement. Handbook of Stress and Burnout in Health Care.

[50] Bakker A.B., Demerouti E., Taris T., Schaufeli W.B., Schreurs P. (2003). A multi-group analysis of the job demands-resources model in four home care organizations. Int. J. Stress Manag..

[51] Bakker A.B., Demerouti E., Verbeke W. (2004). Using the job demands-resources model to predict burnout and performance. Hum. Resour. Manag..

[52] Rapisarda F., Vallarino M., Cavallini E., Barbato A., Brousseau-Paradise C., De Benedictis L., Lesage A. (2020). The Early Impact of the COVID-19 Emergency on Mental Health Workers: A Survey in Lombardy, Italy. Int. J. Environ. Res. Public Health.

[53] Buselli R., Corsi M., Baldanzi S., Chiumiento M., Elena D.L., Dell’Oste V., Bertelloni C.A., Massimetti G., Dell’Osso L., Cristaudo A. (2020). Professional quality of life and mental health outcomes among health care workers exposed to SARS-CoV-2 (COVID-19). Int. J. Environ. Res. Public Health.

[54] Rodriguez-Besteiro S., Tornero-Aguilera J.F., Fernández-Lucas J., Clemente-Suárez V.J. (2021). Gender Differences in the COVID-19 Pandemic Risk Perception, Psychology, and Behaviors of Spanish University Students. Int. J. Environ. Res. Public Health.

[55] Sandín B., Valiente R.M., García-Escalera J., Chorot P. (2020). Impacto psicológico de la pandemia de COVID-19: Efectos negativos y positivos en población española asociados al periodo de confinamiento nacional. Rev. Psicopatol. Psicol. Clín..

[56] Yang X., Zhang B., Wang L., Cao L., Tong R. (2021). Exploring the Relationships between Safety Compliance, Safety Participation and Safety Outcomes: Considering the Moderating Role of Job Burnout. Int. J. Environ. Res. Public Health.

[57] Podsakoff P.M., MacKenzie S.B., Lee J.Y., Podsakoff N.P. (2003). Common method biases in behavioral research: A critical review of the literature and recommended remedies. J. Appl. Psychol..

[58] Signore F., Cortese C.G., Parisi S., Russo V., Zito M., Ingusci E. (2020). Job crafting and well-being at work: An exploratory analysis during health emergency period. Med. Lav..

[59] Nielsen K., Christensen M. (2021). Positive Participatory Organizational Interventions: A Multilevel Approach for Creating Healthy Workplaces. Front. Psychol..

